# Supplementary material for: Exploring the Effect of Water Content and Anion on the Pretreatment of Poplar with Three 1-Ethyl-3-methylimidazolium Ionic Liquids
Source: Molecules. 2020 May 15;25(10):2318. doi: 10.3390/molecules25102318 (PMC7288140; doi:10.3390/molecules25102318)
Supplement: Supplementary file 1 [file molecules-25-02318-s001.pdf]

molecules

MDPI

Supplementary Information

Exploring the Effect of Water Content and Anion on the Pretreatment of Poplar with Three 1-Ethyl-3-methylimidazolium Ionic Liquids

Florence J. V. Gschwend [1], Jason P. Hallett [1] and Agnieszka Brandt-Talbot [1,2,*]

[1] Department of Chemical Engineering, Imperial College London, London SW7 2AZ, United Kingdom; f.gschwend12@imperial.ac.uk (F.J.V.G.); j.hallett@imperial.ac.uk (J.P.H.)
[2] Department of Chemistry, Imperial College London, London W12 0BZ, United Kingdom
* Correspondence: agi@imperial.ac.uk

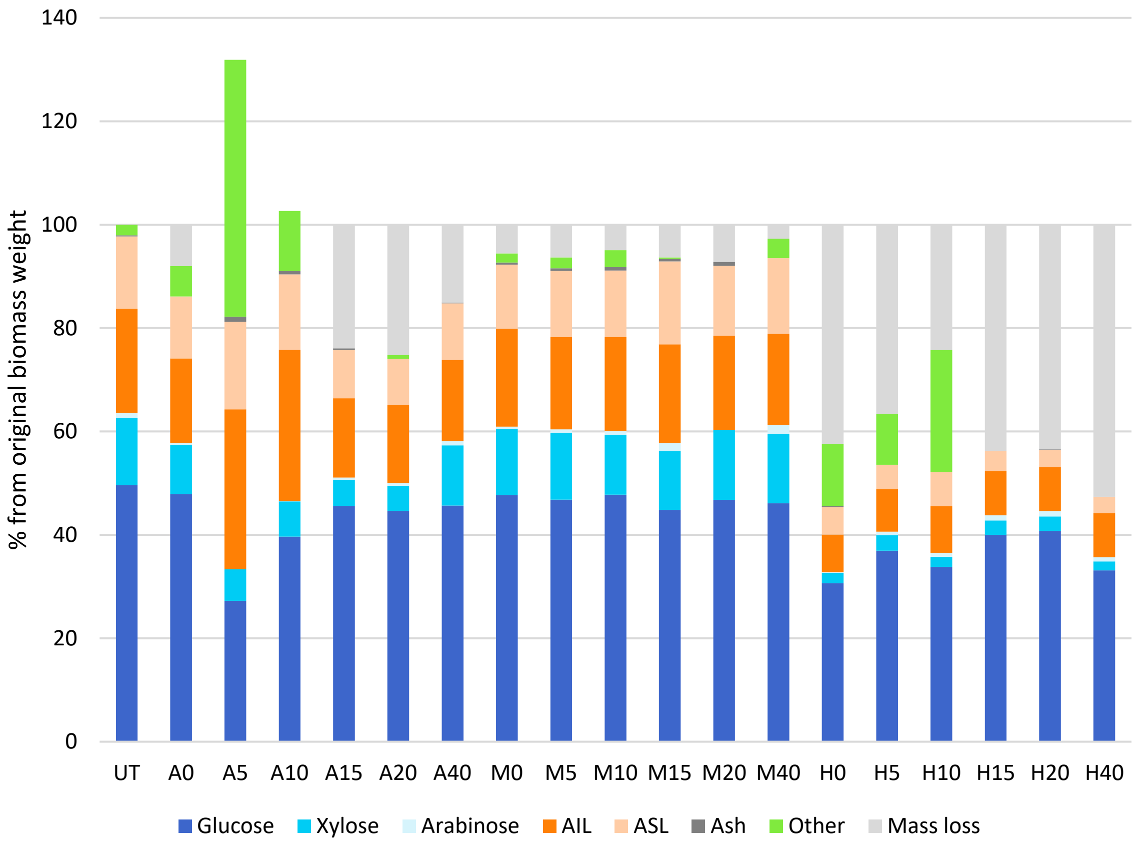

Figure S1. Composition of untreated poplar (UT) and the isolated pulps, adjusted for pulp yields. Other is sample mass that could not be assigned to any structural lignocellulosic component and is likely to be ionic liquid that was not removed during washing of the pulp. Acid insoluble lignin (AIL) and acid soluble lignin content (ASL).

1. Experimental Details

1.1. Moisture Content

Approximately 100 mg of the biomass/pulp was weighed onto a preweighed piece of aluminium foil and the weight recorded using the analytical balance. The foil with the biomass/pulp was folded and oven-dried (T = 105 °C) overnight. The hot packets were placed in a desiccator for 20 min to allow cooling to room temperature. The weight was recorded immediately and the moisture content

calculated. This was performed in triplicate for the untreated biomass and once per sample for the recovered pulp.

1.2. Compositional Analysis

Three-hundred milligrams (on an oven-dried weight (ODW) basis) of air-dried biomass or pulp was weighed out into a 100-mL pressure tube and the weight recorded. Three millilitres of 72% sulphuric acid was added, the samples stirred with a Teflon stir rod, and the pressure tubes placed into a preheated water bath at 30 °C. The samples were stirred again every 15 min for one hour; they were then diluted with 84-mL distilled water and sealed. The samples were autoclaved (Sanyo Labo Autoclave ML5 3020 U) for one hour at 121 °C and left to cool. The samples were then filtered through filtering ceramic crucibles of a known weight. The filtrate was stored in two plastic tubes and the remaining residue washed with distilled water. The crucibles were placed into a convection oven (VWR Venti-Line 115) at 105 °C for 24 ± 2 hours. They were placed in a desiccator for 15 min and the weight recorded. The crucibles were then placed into a muffle oven (Nabertherm + controller P 330) and ashed to constant weight at 575 °C. The crucible weight after ashing was recorded. The content of the acid insoluble lignin (AIL) was determined according to Equation 1:

$$\%AIL = \frac{Weight_{crucible\ plus\ AIR} - Weight_{crucible\ plus\ ash}}{ODW_{sample}} \cdot 100 \tag{1}$$

where $Weight_{crucibles\ plus\ AIR}$ is the weight of the oven-dried crucibles plus the acid insoluble residue, and $Weight_{crucibles\ plus\ ash}$ is the weight of the crucibles after ashing to constant temperature at 575 °C.

The supernatant was used for the determination of the acid soluble lignin content (ASL) by UV analysis at 240 nm (Equation 2) using a Perkin Elmer Lambda 650 UV/Vis spectrometer.

$$\%ASL = \frac{A}{l \cdot \varepsilon \cdot c} \cdot 100 = \frac{A \cdot V_{filtrate}}{l \cdot \varepsilon \cdot ODW_{sample}} \cdot 100 \tag{2}$$

A is the absorbance at 240 nm, l is the path length of the cuvette in cm (1 cm, in this case), ε is the extinction coefficient (12 L/g cm), c is the concentration in mg/mL, ODW is the oven-dried weight of the sample in mg, and $V_{filtrate}$ is the volume of the filtrate in mL and equal to 86.73 mL.

Calcium carbonate was added to the second liquid fraction until pH 5 was reached. The liquid was passed through a 0.2-μm PTFE syringe filter and subsequently submitted to HPLC analysis (Shimadzu, Aminex HPX-97P from Bio-Rad, 300 x 7.8 mm, purified water as the mobile phase at 0.6 ml/min, column temperature 85 °C; de-ashing columns were used as prefilters) for the determination of the total sugar content. Calibration standards with concentrations of 0.1, 1, 2, and 4 mg/mL of glucose, xylose, mannose, arabinose, and galactose were used. Sugar recovery standards were prepared as 10-mL aqueous solutions close to the expected sugar concentrations of the samples and transferred to pressure tubes. Two hundred and seventy-eight microlitres (278 μL) of 72% sulfuric acid was added, the pressure tube closed and autoclaved, and the sugar content determined as described above. The sugar recovery coefficient (SRC) was determined according to Equation 3 and the sugar content of the analysed sample using Equation 4:

$$RC = \frac{c_{HPLC} \cdot V}{initial\ weight} \tag{3}$$

$$\%Sugar = \frac{c_{HPLC} \cdot V \cdot corr_{anhydro}}{SRC \cdot ODW_{sample}} \cdot 100 \tag{4}$$

where c_{HPLC} is the sugar concentration detected by HPLC; V is the initial volume of the solution in mL (10.00 mL for the sugar recovery standards, and 86.73 mL for the samples); the initial weight is the mass of the sugars weighed in; $corr_{anhydro}$ is the correction for the mass increase during hydrolysis of the polymeric sugars (0.90 for the C6 sugars glucose, galactose, and mannose and 0.88 for the C5 sugars xylose and arabinose); and ODW is the oven-dried weight of the sample in mg.

1.3. Enzymatic Saccharification

The ODW basis (100 ± 10 mg) of air-dried pulp was placed into a Sterilin tube and the weight recorded. Three enzyme-only blanks were run with 100 µL of purified water instead of the biomass in order to correct for sugar residues present in the enzyme solutions. A 9.9-mL solution made from 5-mL 100-mM sodium citrate buffer at pH 4.8, 40-µL tetracycline solution (10 mg/mL in 70% ethanol), 30-µL cycloheximide solution (10 mg/mL in purified water), 4.71-mL purified water, and 50 µL of Novozyme's experimental enzyme mixture NS-22201 were added, the tubes closed and placed into an Stuart orbital incubator (S1500) at 50 °C and 250 rpm. After 48 hours, samples were filtered using a PTFE syringe filter. Samples were analysed on a Shimadzu HPLC system with an RI detector and an Aminex HPX-87P column (BioRad, 300 × 7.8 mm) with purified water as the mobile phase (0.6 mL/min). The column temperature was 85 °C, and acquisition time was 40 min. Calibration standards with concentrations of 0.1, 1, 2, 4, and 8 mg/mL of glucose were used.
